# A Few Hints for Young Dentists

**Published:** 1854-07

**Authors:** Geo. L. Paine

**Affiliations:** Xenia, O.


### *A few hints for young Dentists.* By Geo. L. Paine, D. D. S., Xenia, O.

"By persevering you will conquer" is one of the pithy sayings of the noble bard of Mantua.

Having thus announced our text, we ask you to pass over the long list of names "not born to die," because "registered in letters of burning light, on the scroll of fame," and thus learn that in most cases "success is the result of effort"—that by persevering, others have conquered, and by imitating their example, you can likewise conquer; bearing in mind that fame is not inherited, borrowed, nor bought with gold, but that it is the product of patient, plodding, persevering toil.

Confining ourself to the purlieux of our own noble profession, we see an exemplification of the truth of our motto in the career of every eminent Dentist, living or dead—their exalted position being the result of untiring mental and physical labor.

By attending and participating in the periodical meetings of the various associations instituted for the promotion of dental science, mind has been brought in contact with mind, and thus sharpened by the mental attrition, new views have been developed and wholesome suggestions made in relation to important points in dental practice. Under such culture our noble science is improving and will thus continue to improve, until the highest attainable skill and perfection shall be attained.

While some of the devoted and laborious have been busily and patiently engaged in conducting tedious experiments in one department of dentistry, others have been just as actively engaged in different, but equally important departments of our profession. Thus some experimented upon the natural teeth, thus demonstrating their vascularity, their exquisite organization and admirable adaptation to resist the friction of mastication when in health, but when diseased by foul caries, and surcharged with acuteist sensibility, requiring on our part the most careful treatment and delicate manipulation to restore them to health. At the same time, another perhaps was trying to save by a new operation a class of teeth, till then deemed by most, only fit subjects for the dental guillotine—the forceps. While most dentists did not think an aching tooth worth the trouble and labor that might save it, and thus render it a valuable member of the dental community, others thought and acted more wisely, at the same time bending all the energies of their minds to reduce to subjection these refractory subjects which the former would extract, thereby gaining a golden opportunity to insert artificial teeth which in their estimation "pay better."

Though very much has been accomplished during the last quarter century, and though giant strides have been made in every department of dental surgery, a great, a vast deal yet remains to be done ere we as a profession shall occupy that prominent stand-point in the eye of the community—when we shall be regarded as public benefactors, when we shall be able to save a large class of teeth which we must now necessarily extract to be replaced by artificial substitutes, which however well constructed are far inferior to good natural teeth; when the community having undoubted confidence in our skill, shall be more anxious to save their natural teeth and spare neither time nor money to accomplish this important end.

Much, too, remains to be done before artificial teeth shall be so perfected in their adaptation and construction as to be not only good substitutes for the natural teeth, but capable of being worn without injuring and destroying as they now do in most cases the natural teeth to which they are attached. In the experience of all, most partial sets but pave the way for a full set. Now we believe this crying evil might be mitigated, could we in all cases apply partial sets on the atmospheric pressure plan as successfully as full sets. How cheerfully would we dispense with clasps, the great eyesore of mechanical dentistry; and how much our patients would be benefitted by the change!

We feel assured that thousands of teeth are so unskillfully inserted, with so little judgment and taste, that they would be cast aside, as they are in some cases, did not pride or vanity set up its imperious claim to their retention. We ardently hope that some plan will be devised by which these evils may be mitigated or removed entirely; that the brains of thousands now sorely puzzled and taxed to devise some plan, may be relieved and their patients greatly benefitted.

For the attainment of these much desired points, we must look to the efforts of the scientific and laborious portion of the profession who labor in season and out of season for its good, and not to those for whom "Dame Nature has done so much" that scientific research on their part, in their estimation at least, is wholly superfluous. Fortunate souls! they have no need of the teachings of the learned and experienced. Science and its teachings to them is but a clog, a dull routine, an abstraction; like Jonah's gourd they grow up in a night, not "unfledged noviciates" of some Dental college, but fully grown, fully armed and fully prepared, to use their own language, to render entire satisfaction in every department of the profession.

This class of self-taught tooth doctors who are crowding into our ranks are for the most part composed of that class of persons, who, failing in everything in which they may have previously embarked, go into some "one-horse" dental establishment, spend three or four weeks or months, in doing or trying to do, plate-work, and perhaps read "Harris" on filling teeth and plate-work, and forthwith they are dentists, and thus announce themselves to the astonished world, while they are about as competent to put in complete order a delicate patent-lever, or calculate eclipses, by simply understanding the multiplication table, as to assume the responsible and difficult operations entrusted to the care of the dentist.

From this class of self-styled tooth doctors, these kid-gloved soft-handed, soft-headed, book-shunning, college-hating dentists who do the community no good and the profession nothing but evil, and that continually, we may expect nothing, absolutely nothing, in the way of progress, of improvement, or even encouragement to improve.

Whoever, then, that can use his influence to promote the common good, our good, whether by his example, his works, his voice or his pen, should be unsparing in his efforts in this direction; urging as one of the chief means a thorough course of study in some respectable Dental college, prior to assuming the responsibility of practicing on his own hook.

Again, let no dentist hold out inducements to any young man to enter the lists, except he be determined to go through the entire curriculum of collegiate study. If all would thus act we should soon have an intelligent and well educated class of dentists, amply qualified to render service; that a discriminating community would not fail to fully appreciate. Then these prowling dentists, wolves in sheep's clothing, who rely on the cut and quality of their cloth, their white hands and jeweled fingers, or on an extraordinary development of their mechanical bumps, would be compelled for want of bread to retreat to the dark places whence they emerged.

Again, much good may he done, by and through the periodical press and literature of our profession; errors in practice exposed and corrected; judicious advice on all points of practice given; a taste for science instilled, and a laudable ambition for the attainment of excellence inspired.

Great good we think may be occomplished by means of dental associations, designed not to advertise the wares of some mountebank, but for the mutual interchange of thought and feeling on subjects of vital importance to all engaged in dentistry; and we would earnestly urge upon all, especially upon young men just on the threshhold of practice, to attend these meetings as often as possible and there learn the lessons of experience as given by the savans in our profession. No week in the year could be so profitably and judiciously spent by the young practitioner, as the one set apart for these meetings.

By pursuing the course we have feebly indicated, we can see no reason why any young man should not attain to respectabiltty as a dentist. Let him glean rich sheaves of knowledge from the whole domain of medical and dental literature; let him be on good terms with the learned and skillful, the experienced and reputable; and let him ever be ready with his pen, his voice and his influence to advocate and urge the necessity of a thorough dental education; let him, in a word, be wedded to his profession, making it the child of his love, and our word for it, he will enroll his name high on the list of those "whose names were born not to die."

In fine, let the young dentist be more ambitious to do well and thoroughly whatever he undertakes, than to try to accomplish a great deal in a short time, when as a necessary result, his operations must be imperfect.

"As you sow, so shall ye reap," is as true in dentistry as in morals, and as you do your work now, so will be your future practice, your reputation, your wealth.

XENIA, O., Jan., 1854.

